# Immune mechanisms associated with cardiovascular disease in systemic lupus erythematosus: A path to potential biomarkers

**DOI:** 10.3389/fimmu.2022.974826

**Published:** 2022-11-07

**Authors:** Gabriela Guzmán-Martínez, Concepción Marañón, CYTED RIBLES Network, Ana María Blasini

**Affiliations:** ^1^ Atrys Health, Madrid, Spain; ^2^ Department of Medicine, Faculty of Biomedical and Health Sciences, Universidad Europea de Madrid, Madrid, Spain; ^3^ Department of Cardiology, La Paz University Hospital, IdiPaz, Madrid, Spain; ^4^ Department of Genomic Medicine, Pfizer-University of Granada-Andalusian Regional Government Centre for Genomics and Oncological Research (GENYO), Granada, Spain

**Keywords:** systemic lupus erythematosus, cardiovascular disease, inflammation, cytokines, autoantibodies, biomarkers

## Abstract

Systemic lupus erythematosus (SLE) patients display an increased risk of cardiovascular disease (CVD). With the improved clinical management of other classical severe manifestation of the disease, CVD is becoming one of the most relevant complications of SLE, and it is an important factor causing morbidity and mortality. Several immune constituents have been shown to be involved in the pathogenesis of atherosclerosis and endothelial damage in SLE patients, including specific circulating cell populations, autoantibodies, and inflammatory mediators. In this review, we summarize the presentation of CVD in SLE and the role of the autoimmune responses present in SLE patients in the induction of atherogenesis, endothelial impairment and cardiac disease. Additionally, we discuss the utility of these immune mediators as early CVD biomarkers and targets for clinical intervention in SLE patients.

## Introduction

**Figure 1:**
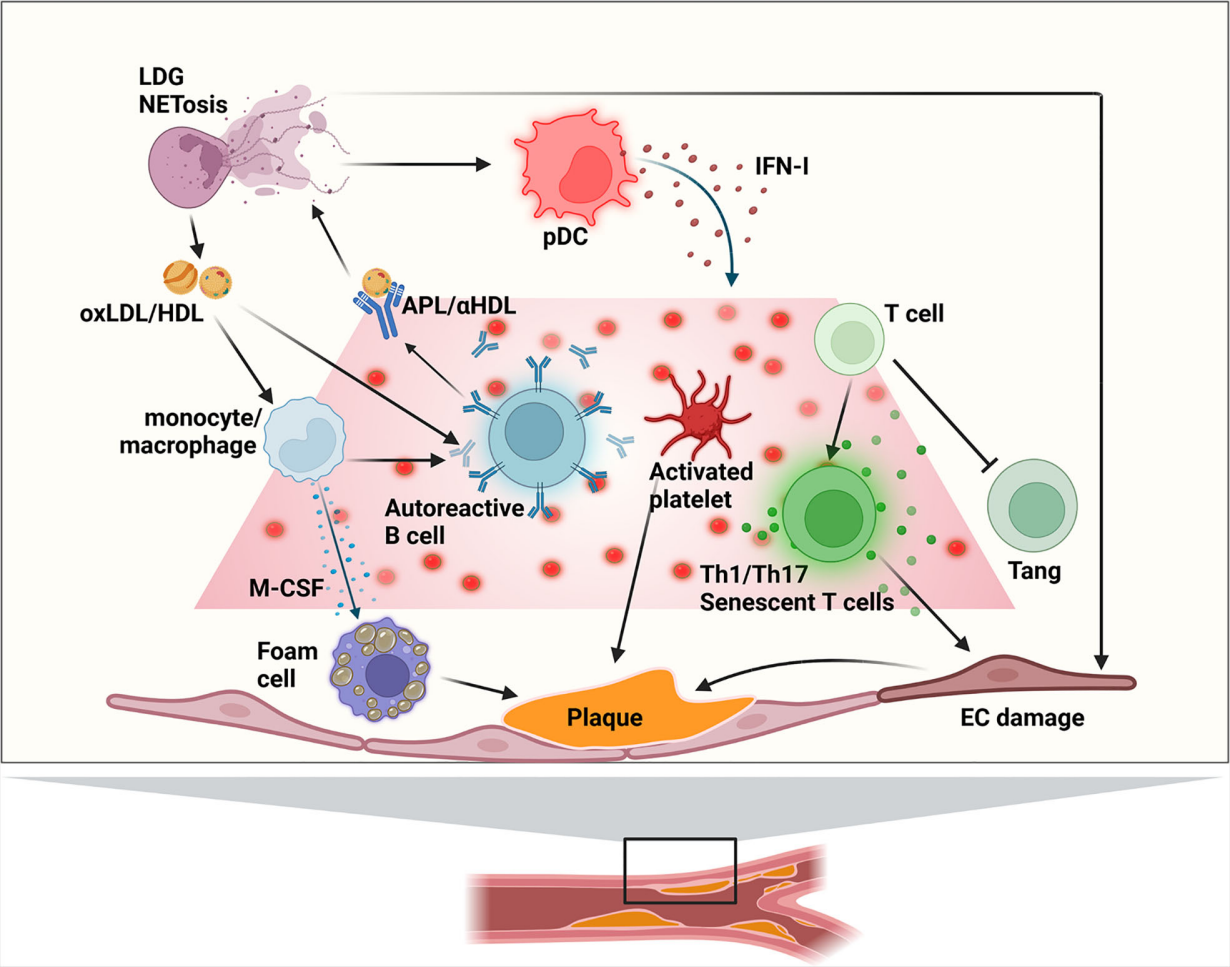
Main immune mechanisms of involved atherogenesis in SLE patients. APL, anti-phospholipid antibody; EC, endothelial cell; (ox)HDL, (oxidized) high-density lipoprotein; IFN-I, type I interferon; LDG, low-density granulocytes; (ox)LDL, (oxidized) low-density lipoprotein; pDC, plasmacytoid dendritic cell; Tang, angiogenic T cell.

The prevalence of cardiac diseases in systemic lupus erythematosus (SLE) is reported to be higher than 50% at some point in the patients’ life (1). Growing evidence shows that the immune system has a significant influence on the generation of the atherosclerotic plaque and cardiovascular disease (CVD). SLE is a heterogeneous autoimmune disease associated with significant morbidity and mortality. In the ‘70s, a bimodal mortality peak for lupus patients was described; the first one was attributed to secondary infections and tissue damage and the second to CVD events (2). Thirty years later, current progress in disease management has resulted in a decrease of mortality due to disease activity; however, CVD events and infections remain major mortality causes (3). The traditional risk factors associated with atherosclerosis like smoking, diabetes, increased body mass index (BMI), dyslipidemia or hypertension, are also present in SLE patients. However, the high rates of ischemic events observed so far cannot be explained by the standard Framingham scores (4), since the atherosclerotic process is accelerated in SLE patients due to a complex interaction of traditional and inflammatory mechanisms (**Figure 1**) (5–7). Moreover, SLE itself is considered an independent risk factor for endothelial dysfunction (8). Consequently, other scores have been published with the aim of better measuring CVD risk specifically in SLE patients: Urowitz et al. proposed a risk score for a broad class of cardiovascular events derived by simply multiplying the components of the Framingham risk score by 2 (9). The SLE cardiovascular risk score derived by Petri et al, identified both traditional cardiovascular and SLE-related risk factors, including global activity score (the SELENA-SLEDAI score), low C3 and the lupus anticoagulant (10). The QRISK3 score was designed to address CVD risk associated with SLE and, apart from the presence of SLE, it includes the following items: chronic kidney disease, migraine, severe mental illness, atypical antipsychotic use, corticosteroid use, erectile dysfunction and systolic blood pressure variations over time (11). Finally, the global APS score (GAPSS) (12) and its adjusted version (13) was designed to bring improvement in risk prediction of thrombosis by scoring traditional risk factors such as hyperlipidemia and arterial hypertension, in combination with antiphospholipid antibodies (lupus anticoagulant, anti-cardiolipins, anti-β2-glycoprotein I and anti-phosphatidylserine-prothrombin). All these scores specifically constructed to evaluate risk of CVD in SLE still require independent external validation before being widely used in the clinical practice.

The prevalence of ischemic heart disease in SLE patients is estimated between 3.8% and 16%, depending of the study (3, 14). This is a 10-fold risk compared to the general population, and a 50-fold risk in young women at reproductive age (4). Different studies showed an increase of 2 to 8-fold in the risk of stroke in SLE patients (4, 15, 16). There is evidence of subclinical atherosclerosis lesions in 30–40% of patients with SLE, which varied according to the method of diagnosis used. The carotid plaque thickness in SLE patients is particularly unusual in those patients under 55 years old and several reports show that SLE patients have higher prevalence of atherosclerotic plaques compared with healthy donors (3, 5). In a meta-analysis, SLE patients had 2-fold prevalence of carotid plaques compared with matched controls (17). A longitudinal study showed that SLE women with carotid plaque at baseline had a significant increase in the incidence of CVD during an 8 years follow-up (18).

## Cardiovascular manifestations in SLE

**Figure 2:**
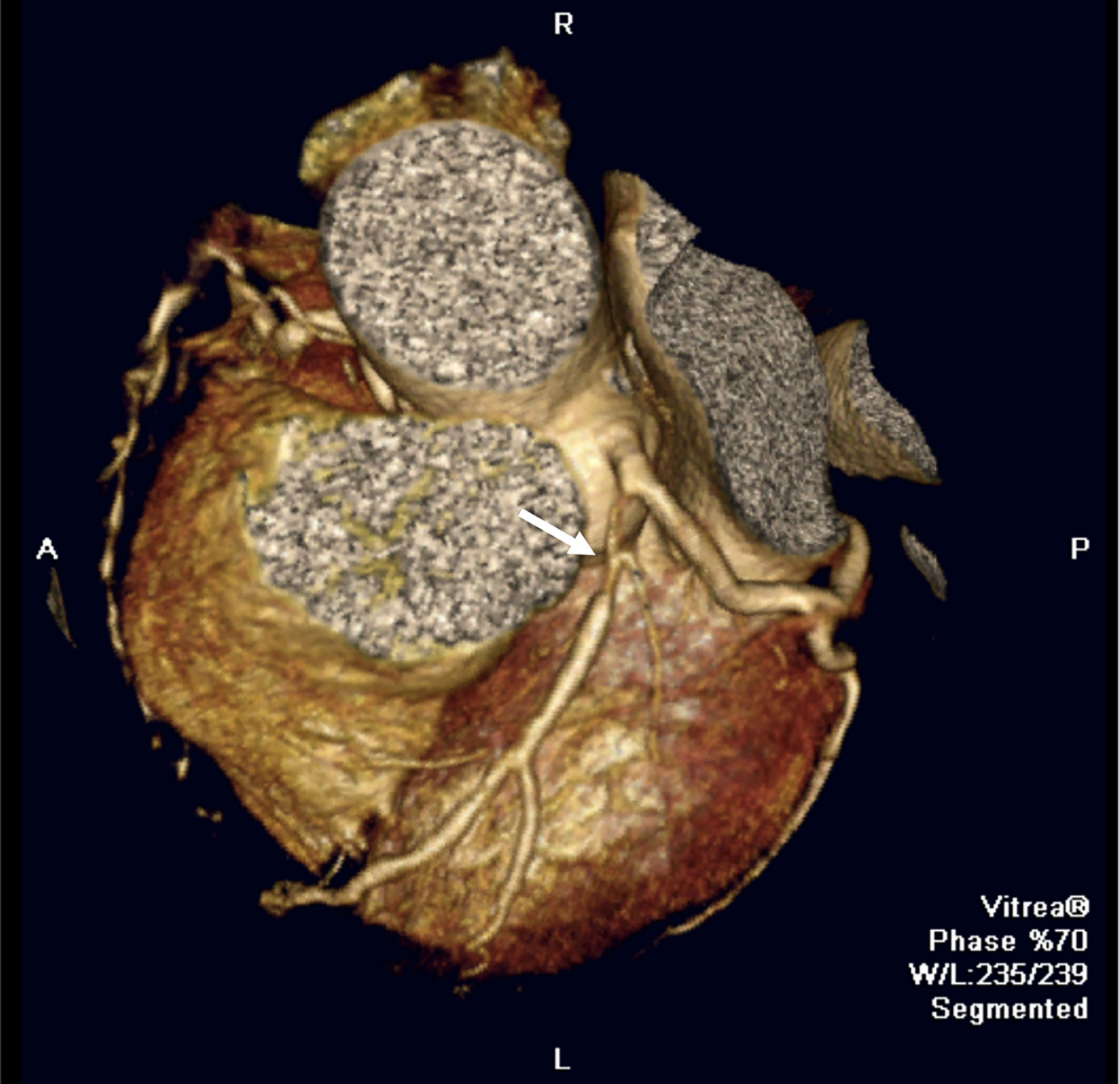
Cardiovascular manifestations in SLE. Coronary computed tomography image showing a severe stenotic lesion in the proximal segment of the left anterior descending coronary artery (white arrow) in a SLE patient.

Cardiac involvement in patients with SLE can negatively impact all components of the cardiovascular system and heart, including the valve endocardium, myocardium, pericardium, conducting system and coronary arteries. Thus, the main cardiological or cardiovascular manifestations that we can find in SLE are: endocardial involvement (endocarditis) (19), myocardial involvement (subclinical myocardial involvement and lupus myocarditis) (20), pericardial involvement (pericarditis, pericardial effusion, and tamponade) (21), conducting system involvement (bradiarrytmias, taquiarrytmias, long QT syndrome) (22) and coronary artery disease (CAD). In the field of diagnosis, systematic screening for coronary disease in asymptomatic patients is not established in the clinical practice. In symptomatic patients, echocardiography, ischemia induction tests, noninvasive coronary angiography using computed tomography (**Figure 2**), and invasive coronary angiography using cardiac catheterization are recommended.

Coronary disease is the cardiac disorder with higher correlation with immunological parameters in SLE. The type of condition in patients with SLE can vary and three types of pathology can be found: thrombosis/embolization of the lumen, inflammation of the vascular wall and coronary atherosclerosis. Most published cases of myocardial infarction (MI) in patients with SLE are due to the presence of coronary atherosclerosis. This condition is more frequent in male patients, associated with older age and longer duration of the disease (23). As for the general population, subclinical atherosclerosis is also more prevalent than clinical CVD in SLE patients. But, in addition, it is also more prevalent than in subjects without SLE. In an autopsy series, coronary atherosclerosis was observed in up to 40% of SLE patient segments (24). The risk of clinical CVD in patients with SLE is highly variable depending on the studies analyzed, and is around 2-10 times higher than that in the general population, even after adjusting for traditional cardiovascular risk factors (4). In another study that included 4,863 people with SLE, the adjusted HR for MI was 2.61 compared to controls without SLE (15). This risk increased to 5.6 during the first years after diagnosis, probably due to the role of the active inflammation. Furthermore, it is interesting to note that premenopausal women with SLE are around 50 times more likely to have a MI infarction than sex and age-matched controls (25). Regarding racial and ethnic distribution, in a recent study including 65,788 cases of SLE, there was a reduced risk of MI among Hispanics and Asians compared to Caucasian patients with SLE (HR: 0.61 and HR: 0.57, respectively) (26), similarly to what is observed in subjects without SLE.

Poorer outcomes are also observed in the evolution of long-term ischemic heart disease, including mortality. Thus, although post-infarction in-hospital results regarding the need for revascularization (both percutaneous and surgical) do not differ between patients with SLE and controls (27, 28), a significant difference has been observed in long-term out-of-hospital results. Accordingly, patients with SLE are more likely to experience a new MI or to need a second percutaneous intervention in the year following the initial event (29). The mortality rate is also affected, since SLE patients with MI are at least 2.6 times more likely to die than non-SLE patients with the same coronary event (30).

## Traditional and non-traditional risk factors of CVD in SLE

Metabolic syndrome is the result of a combination of central obesity, insulin resistance, dyslipidemia and hypertension. The prevalence rates rank from 15.8% to 32.4% vs 4.2% to 10.9%, in SLE patients when compared to age-matched healthy donors (31, 32). In SLE patients the presence of metabolic syndrome has been associated with the following factors: increasing age, racial/ethnic ancestry (mainly Hispanic or Black African), disease-related characteristics such as baseline renal disease, Systemic Lupus International Collaborative Clinics damage index (SDI) >1, higher disease activity, coronary atherosclerosis, arterial stiffness and inflammatory biomarkers (3). Increased BMI was significantly associated with subclinical atherosclerosis in SLE populations (33). Insulin resistance also occurs more often in SLE patients, associated with higher BMI, SDI, hypertension and corticosteroid prescription (34).

Arterial hypertension is a recognized risk factor for CVD (35), and is present in 33-74% of SLE patients (36, 37) and is a recognized risk factor for CVD development in SLE patients (35). A longitudinal study investigated the determinants of atherosclerosis progression in 187 SLE patients, detecting age and hypertension as being independent factors associated with the progression of carotid intima-medial thickness (IMT) and plaque formation (38). Renal disease, insulin levels and SLE disease activity index (SLEDAI) have also been reported as independent predictors of hypertension in SLE (36). The night-time blood pressure patterns (steady, non-dipping hypertension or nocturnal hypertension/reverse dipping) in women with SLE were assessed in a subsequent study, showing that these patterns were more frequent in SLE and independently associated with increased carotid-femoral pulse wave velocity (37).

High levels of total cholesterol and low-density lipoprotein (LDL), combined with low levels of high-density lipoprotein (HDL), are associated with increased risk for CVD in SLE (3, 8). Dyslipidemia in SLE patients range from 36% to more than 60% within a three year of follow up (38). The classical pattern found in these patients is characterized by increased levels of very-low-density lipoproteins (VLDL), triglycerides and low levels of HDL, which can be worsened by disease activity (39). Besides, SLE patients have frequently increased levels of atherogenic small dense LDL particles (40). Similarly, circulating lipoprotein remnant particles and the intermediate density lipoprotein (IDL) fraction have also been strongly associated with IMT in SLE patients, while small HDL particles have been associated with activation of the complement system, linked with higher IMT values (41). A proinflammatory HDL subtype (piHDL), is also detected in a high proportion of patients with SLE, and is associated with carotid artery plaque and clinical CVD (3). Finally, in SLE patients, there are higher highlipoprotein(a) [Lp(a)] levels compared to subjects of the same sex and age, and these increased Lp(a) values are independent predictors of atherosclerosis (42, 43).

Smoking has been associated with CVD, cerebrovascular and peripheral vascular events in SLE (44), being identified as a risk factor for progression of coronary artery calcification, independent of gender, age, or ancestry (38).

Hyperhomocysteinemia is found in 11-81% of SLE patients versus 0.8-20% in healthy controls, showing an association with subsequent development of CAD, thrombotic effects and markers of subclinical atherosclerosis (45).

Diverse factors such as high anti-phospholipids (APL) autoantibody titers, impaired renal function, low leukocyte cell count, lymphopenia and renal disease have been associated with carotid IMT and arterial stiffness (46, 47). The formation of the carotid plaques may happen twice as frequently in SLE patients with lupus nephritis (LN) compared to age-matched non-nephritis SLE patients and healthy controls, mainly in hypertension patients (46). Disease duration, high SDI chronicity scores and disease activity were identified as important factors for CVD development in SLE (47, 48). Duration of the disease has also been independently associated with coronary artery calcification and carotid plaque formation and progression. In addition, the SDI score was found to be independently associated with clinical CVD, increased IMT, carotid plaque formation, and arterial stiffness (3, 49).

## Cytokines and CVD in SLE

**Figure 3:**
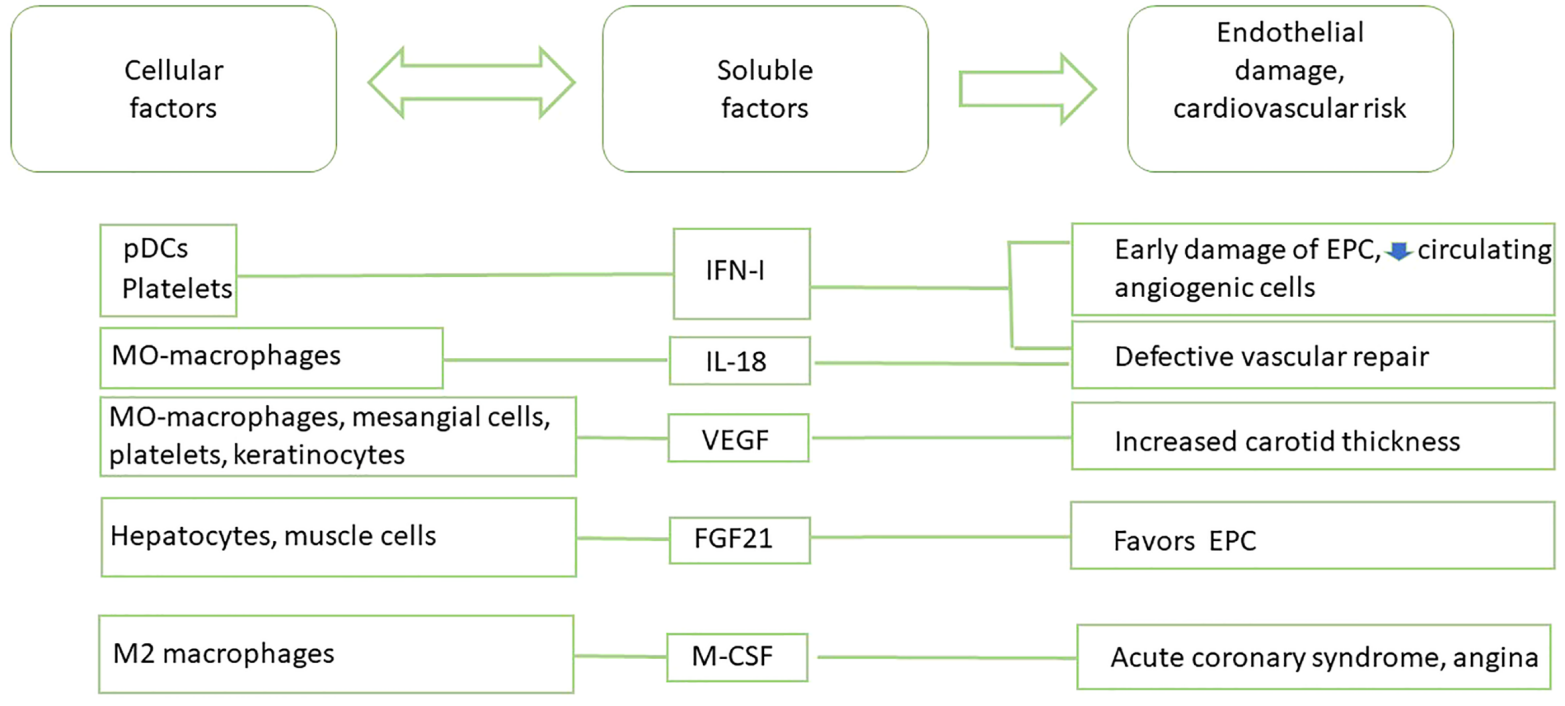
Main cytokine-based biomarkers of CVD in SLE patients.

New knowledge on the complex pathways linking core abnormalities in the innate and adaptative branches of the immune response and endothelial cell (EC) function has broadened our comprehension of the accelerated vascular damage occurring in SLE (**Figure 1**). Cytokines are important modulators of smooth muscular cell activity and death, cell proliferation and monocyte/macrophage localization, mediating plaque growth and generation of the fibrous cap. Moreover, cytokines can determine the stability of the atheromatous plaque (50). Cytokines participating in inflammatory processes can have a role in the early presentation of atherosclerosis in SLE, but also the inflammatory response induced by cytokines in EC and macrophages are important (**Figure 3**).

The main cytokine positively correlated with CVD in SLE is type I interferon (IFN-I, mainly IFNα and IFNβ). It dysregulates neutrophil function, and induces changes in cell metabolites that are emerging as important regulators of systemic immune dysfunction and as strong risk factors for premature CVD in SLE. Accumulative evidences have widened the role of IFN-I in disease, from antivirus defense to autoimmune responses and immuno-metabolic syndromes. The significant pathogenetic role of IFN-I in several systemic autoimmune diseases including SLE is now well recognized. Elevated circulating IFN-I level is associated with CVD in patients with different interferonopathies. Additionally, experimental data have attested that IFN-I affects plaque-residing macrophages, potentiating foam cell and extracellular trap formation, inducing endothelial dysfunction, and altering the functionality of dendritic cells (DC) and T and B lymphocytes. All these immune-pathological mechanisms lead to exacerbated atherosclerosis outcomes and insulin resistance (51, 52). Recent studies have also discovered a relationship between skewed IFN-I responses and metabolic disorders. IFN-I responses to self-nucleic acid-driven Toll-like receptor (TLR) activation in plasmacytoid dendritic cells (pDC) is the key initiating event shared by autoimmune and metabolic diseases (53). Interestingly, activation of IFN signature has also been described in platelets of SLE patients with a history of CVD, suggesting that the presence of platelets with IFN-I signature could be a novel marker for CVD in SLE (54).

It has been reported that the elevated levels of IFN-I associated with SLE alter the balance between vascular damage and repair, thus promoting CVD phenotype (55). Accordingly, an elevated serum IFN-I activity was associated with decreased endothelial function and severity of coronary calcification in SLE patients, even after correction for traditional CVD risk factors (56). IFN-I promotes early atherosclerosis in SLE, inducing an abnormal phenotype and function of endothelial progenitor cells (EPC) and circulating angiogenic cells (CAC), which are crucial for vessel repair after a vascular damage. IFNα induces the apoptosis of EPC and CAC and polarizes myeloid cells towards a non-angiogenic phenotype. Strikingly, neutralization of IFN-I pathway restored a normal EPC/CAC phenotype (57). The detrimental effects of IFN-I on vasculogenesis in SLE could also be mediated by repression of vascular repair mediated by the IL-1 pathway. IFNα represses mediators such as IL-1α IL-1β, IL-1R1, and VEGF, and upregulates IL-1RA and the decoy receptor IL-1R2. Of note, IL-1β promotes significant improvement in the functional capacity of lupus EPC/CAC (58). The urinary levels of vascular endothelial growth factor (VEGF) have been evaluated as a biomarker of LN. Its role in fibrosing diseases is clear and VEGF inhibition has been used as a therapeutic tool (59). VEGF plasma levels have been also associated with disease activity, higher mean carotid IMT, and could be a novel cardiovascular risk factor in premature coronary atherosclerosis in SLE (60, 61). However, serum IL-18, which is also processed by the inflammasome as the IL-1 familiy is elevated in SLE patients and correlates with EPC/CAC dysfunction. Exogenous IL-18 inhibits endothelial differentiation in EPC/CAC, supporting a negative effect of IL-18 on vascular repair *in vivo* (62). Thus, the effects of IFN-I are complex and can contribute to an elevated risk of CVD through diverse mechanisms. Interestingly, treatment of SLE patients with anifrolumab (bloking IFN-I receptor) significantly reduced NETosis and TNFα levels, improving also cardiovascular profiles (63)

Other cytokines have been reported as biomarkers associated with CVD in SLE, while with a lower level of evidence. Among them, fibroblast growth factor 21 (FGF21) and epidermal growth factor receptor (EGFR), which binds multiple EGF ligands, have multiple functions that modulate vascular smooth muscle cells, cardiomyocytes, cardiac fibroblasts, EC, adipocytes, and immune cells (64). However, a recent study found no significant differences in EGF levels in SLE patients with CVD or showing atheromatous plaques (65). FGF21, a liver-secreted protein, plays a crucial role in glucose homeostasis and lipid metabolism. FGF21 has been reported to attenuate the progression of atherosclerosis, but its impact on endothelial progenitor cells under high oxidative stress is not clear, and no evidence exits about the changes of levels of this cytokine in SLE. Anyhow, FGF21 could be a promising biomarker, since its reduction could be associated with low levels of these progenitor cells in SLE (66).

M-CSF is an important cytokine for the differentiation and phenotype of monocytes and macrophages, and is a marker of M2 macrophages. M-CSF can be produced by activated macrophages, lymphocytes and mesenchymal cells (66, 67), and is one of the strongest risk factors for adverse outcomes and an indicator of acute coronary syndrome in patients with stable angina. M-CSF levels were significantly elevated in patients with ACS compared with patients with stable angina, probably due to smooth muscle cell loss caused by the activation of metalloproteinases in the plaque. Serum M-CSF levels 6 weeks after discharge in patients with severe unstable angina were associated with of cardiac events during a 2-year follow-up (68). Recently M-CSF has been evaluated as a biomarker of disease activity and renal involvement in SLE, with the higher levels being predominantly derived from monocytes. These data highlight the potential value of M-CSF as biomarker in the clinical management of SLE patients (67), although it is not clear yet if in SLE patients with isolated atherosclerosis, the levels of M-CSF are a reliable biomarker of adverse outcomes in cardiovascular events.

Several evidences of the implication of pro-inflammatory cytokines in CVD have been also reported (69), sometimes with contradictory outcomes. For instance, high serum IL-6 concentration was described as an atherosclerotic risk marker in several cohorts (70, 71), but not in others (7, 72). High plasma TWEAK levels were strongly associated with plaque in SLE women with higher odd ratios than piHLD (73). It is noteworthy that TWEAK has also been described as a biomarker of LN (74). A positive correlation between serum TNFα and cardiovascular risk in children with SLE has been described (75). TNFα and BAFF were also associated with CVD in adults with SLE (76). Of note, high BAFF was associated with subclinical atherosclerosis, and it has been suggested that the anti-BAFF biologics belimumab could induce IMT decrease in SLE patients with mid-low body mass index (BMI). Moreover, the use of TNF-targeting drugs is associated with a reduction of MI and cardiovascular events in rheumatoid arthritis patients (77). Accordingly, the positive correlation between BAFF and internal carotid artery thickness was lost in SLE patients with high BMI (78). Finally, an *IL19* risk allele has been associated with stroke/MI in SLE and rheumatoid arthritis. The risk allele affects the binding of transcription factors to the locus, and the expression of the IL-10 protein, coded in the same locus. Moreover, *IL19* risk allele was associated with higher APL titers in SLE patients (79).

## Autoantibodies

The accelerated atherosclerosis evolution observed in SLE patients is characterized by an endothelial involvement inducing the development and progression of atheromatous plaques, stimulation and activation of EC and recruitment of neutrophils in the areas affected (80). This results from the activity of the innate and acquired immune responses, as well as the presence of autoantibodies and immune complexes (see **Table 1**; **Figure 1**). One of the hallmarks of SLE is the production of autoantibodies to several autoantigens. Among them, APL (anticardiolipin (CL), anti-β2-glycoprotein 1 (anti-β2GP1) and lupus anticoagulant)have been extensively studied, with different reports detecting them in 20-30% of patients with SLE. They have been associated with a higher risk of atherosclerosis and cardiovascular events in SLE patients in a cohort of more than 600 individuals (81), as well as in the general population (81–83). APL may exert such effects through different potential mechanisms; for example, high expression of β2GP1 in monocytes has been reported in SLE and antiphospholipid syndrome, and proliferative responses to it correlate with the internal carotid artery thickness and with a history of arterial thrombosis (84). Furthermore, anti-β2GP1 induces the assembly of inflammasomes in the EC and the release of endothelial vesicles enriched in mature IL-1β. These cells have a distinct miRNA profile and cause EC activation. In turn, EC-derived extracellular vesicles activate unstimulated EC through a pathway dependent of TLR7 and ssRNA. The alterations in miRNA content may contribute to the ability of these endothelial vesicles from EC cells exposed to anti-β2GP1 to activate unstimulated EC in an autocrine and paracrine manner (85). Additionally, anti-β2GP1 can activate EC through TLR4 (86). Furthermore, anti-β2GP1 increase the expression of cell adhesion molecules, including E-selectin, vascular cell adhesion molecule-1 (VCAM-1) an intracellular adhesion molecule-1 (ICAM-1) and this could increase the attraction of monocytes (87). Additionally, β2GP1forms stable and non-dissociable complexes with oxidized LDL (oxLDL), and they are recognized by IgG anti-β2GP1 autoantibodies, facilitating macrophage-derived foam cell formation (88). In animal models, the oxLDL/β2GP1/anti-β2GP1 complexes increase foam cell formation, TLR4 expression, NF-κB activation, tissue factor expression, and TNFα and MCP-1 secretion (89). β2GP1 expressed within the subendothelial regions and intima-medial borders of atherosclerotic plaques causes specific T-cells reactivity, with a role in fatty streak formation (90). Finally, as β2GP1 inhibits von Willebrand factor activity, and thus anti-β2GPI would induce thrombosis (91).

**Table 1:** Mechanism of action of autoantibodies in SLE-associated CVD.

Autoantibody specificity	Endothelial action	Atherosclerotic/atherogenic action
Antiphospholipid	⇑	⇑
Anti-dsDNA	⇑	⇑
AECA^(1)^	⇑	
Anti-HDL		⇑
Anti ApoA-I		⇑
IgG anti-oxLDL		⇑
IgM anti-OxLDL		⇓
Anti Lp(a)		⇑
IgM anti-phosphorylcholine		⇓
IgM anti-malondialdehyde		⇓

^(1)^AECA, anti-endothelial cell antibodies; arrow up: favours, arrow down: negative regulation.

Anti-dsDNA antibodies are associated with aberrant activation of innate immune cells in particular monocytes and neutrophils. They induce NETosis in neutrophils, apoptosis in monocytes, and modulate inflammation, thrombosis-related molecules and EC activation (92). Oxidant-generating enzymes, generated by NETosis, would oxidize HDL, modifying it to a proatherogenic lipoprotein (93). Patiño-Trives et al. evaluated 85 patients with SLE, finding that the presence of anti-dsDNA antibodies are associated with endothelial dysfunction, proatherogenic dyslipidemia and accelerated atherosclerosis. The authors suggested an alteration of key molecular processes that drive a distinctive and coordinated immune and vascular activation, driving an increase in cardiovascular risk (92).

Anti-EC antibodies (AECA) is a group of antibodies directed against EC proteins. The prevalence of AECA in patients with SLE ranges between 15 and 88% (94). These immune complexes have been associated in SLE patients with vasculitis, inducing the release of proinflammatory factors and adhesion molecules through the activation of NF-kB. This leads to the release of E-selectin, ICAM-1, VCAM-1, cytokines (IL-1, IL-6, IL-8) and chemokines (MCP-1) (95).

IgG autoantibodies against HDL and apoliprotein A-I (ApoA-I) are increased in SLE patients in a study including 77 SLE patients and paired controls, showing that the presence of IgG anti-HDL and Apo A-I produced destabilization of the atheromatous plaque (96). Similar results were reported in other cohorts (97). A dual effect of HDL has been described: it can be anti-inflammatory in basal state and pro-inflammatory (piHDL) in states of acute phase response in a study comparing 154 SLE, 48 rheumatoid arthritis and 72 controls (98). The protective effect of HDL depends largely on the content of apoA-I that mediates the union with macrophages. ApoA-I can become immunogenic, inducing antibodies that modify myeloperoxidase in neutrophils, leading to a destabilization of atheromatous plaques (99). These antibodies would be generated due to the protein misfolding stimulated by the oxidative microenvironment. The misfolded and oxidized ApoA-I is likely to be more immunogenic, leading to higher titers of anti-ApoA-I and probably anti-HDL (100). These antibodies correlate with a lower paraoxonase activity (96), which is associated with subclinical atherosclerosis (101). Furthermore, anti-HDL and anti-ApoA-I could cross-react with anti-CL, which in turn cross-reacts to HDL and less frequently with ApoA-I. The frequency of these antibodies in patients with SLE and APS fluctuates between 7.7% and 32.5% (102). O’Neill et al. described the association of these antibodies with SLE activity, and this may support the accelerated development of atheromatous plaques in patients with inflammatory disease, such as SLE, during active clinical activity (103). Oxidized LDL (oxLDL) has chemotactic, immune-stimulating properties and the ability to be taken up by macrophages in atheromatous plaques, inducing their differentiation to foam cells (104). Interestingly, anti-oxLDL has been reported in more than 50% of SLE patients (105). Lopez et al. found that patients with SLE with increased carotid IMT (n=30) had elevated levels of IgG-oxLDL/β2GP1 immune complexes (106). IgG anti-oxLDL is associated with atherosclerosis, but IgM anti-oxLDL seems to be protective (107). Antibodies against the oxidized fraction of Lp(a) have been found in patients with SLE and antiphospholipid syndrome, and could be a way of producing atherosclerosis (108), although more clinical and *in vitro* studies must be carried out to determine their predictive value.

IgM anti-phosphorylcholine has a cardio-protective mechanism in the general population, and also in SLE patients. The effect of IgM anti-phosphorylcholine seems to be mediated by its effect on the reduction of pro-inflammatory and pro-atherogenic T lymphocytes, and the increase of Tregs, keeping dendritic cells in an immature stage, potentially tolerogenic (109, 110). These antibodies seem to be involved in the clearance of apoptotic cells, and their decreased levels could be related to a higher burden of apoptotic cells or an immune dysfunction, leading to a decreased production of protective antibodies in SLE patients (111). High triglyceride and low HDL are associated with a low IgM anti-phosphorylcholine level (109). Furthermore, low levels of IgM anti-phosphorylcholine and IgM anti-malondialdehyde have been associated with plaque occurrence in SLE (112, 113).

## Immune cells

Recent discoveries about the role of innate and adaptive immune cells in SLE immunopathology and mechanisms cross-targeting EC has greatly contributed to our understanding of the abnormalities leading to CVD in SLE patients. Increased proportions of pro-atherogenic CD16^+^ monocytes, low-density granulocytes (LDG), Th17 cells and senescent CD4^+^CD28^null^ lymphocyte subsets, along with reduced numbers of vascular repairing endothelial progenitor cells (EPCs) and angiogenic T cells, all contribute jointly to the development of atheromatosis in SLE patients. This new knowledge may set the basis for the development of novel cell biomarkers allowing earlier identification and opportune preventive measures of CVD risk associated to SLE (see **Box 1**).

Box 1 **Candidate cellular biomarkers of cardiovascular risk in SLE patients.**

- Detection and quantification of NETs by flow cytometry as a marker of enhanced endothelial damage in SLE (114).
- Measurement of MMP-9 and of MMP-9/anti- MMP-9 complexes in serum (115).
- Genomic microarrays to identify LDGs with increased potential for inducing endothelial damage in SLE patients (116).
- Proteomic analysis of LDG to test for citrullinated H3, a known marker of NETosis in sepsis and cancer (117), as well as other epigenetically modified neutrophil components potentially exacerbating endothelial damage.
- Measurement of circulating EPC as a cell marker of subclinical atherosclerosis in SLE (118).
- Measurement of CD14^dim^CD16^+^ (non-classical monocytes) as cell marker related to IMT in SLE patients (119).
- Monocyte to HDL ratio (MHR) as a biomarker of systemic inflammation, subclinical cardiovascular risk, cardiovascular risk in chronic kidney disease (120–122).
- CD8^+^ Tang cells as a biomarker of endothelial damage and lupus nephritis relapse (123, 124).
- CD8^+^Tang cells + anti-dsDNA as markers of endothelial damage (125).
- CD4^+^CD28^-^ T cells as markers of immunosenescence, chronic inflammation and endothelial damage (126).

The original report of an expanded population of LDG in patients with SLE (127) increased our understanding of the role of innate immune mechanisms in SLE. These cells were increased in SLE patients (n=64), and independently of Framingham scores they associated with vascular inflammation and coronary disease (128). LDG show an increased propensity to produce neutrophil extracellular traps (NETs), a modality of cell death characterized by the extrusion of modified chromatin and cellular anti-microbial proteins used by granulocytes to fight infectious agents (129). Compared to normal LDG, LDG of SLE patients have a strong pro-inflammatory signature (128) and are less able to circulate in the microvasculature (130), rendering them more likely to adhere and damage EC (115). Incubation of HUVEC with LDG-derived NETs from SLE patients *in vitro* induces pronounced morphological changes suggestive of endothelial damage, as compared to normal density granulocyte-derived NETs from healthy controls. The functional relevance was demonstrated in thoracic aorta rings, showing a more prominent impairment of vasodilation when exposed to LDG-derived NETs compared to normal density granulocyte-derived NETs. In addition, MMP-9 metalloproteinase is activated and externalized during NETosis which, in turn activates endothelial MMP-2 (115).

Cholesterol microcrystals induce NETosis in the early stages of vessel plaque formation, a process accelerated by pro-inflammatory cytokines produced by TLR2- and TLR4-stimulated macrophages (129). In addition, some NETs products, such as myeloperoxidase, can oxidize LDL and HDL, generating pro-atherogenic compounds interfering with cholesterol efflux from macrophages in the subintima of arterial walls (93). In addition, several metalloproteinases are activated and externalized during NETosis; endothelial MMP-2 is activated by MMP-9 present in NETs (115). Oxidized mitochondrial components present in NETs are potent inducers of IFN-I in pDC (131–133), by a mechanism involving activation of the cyclic GMP-AMP synthase (c-GAS)-stimulator of interferon genes (STING) pathway (131). The STING pathway contributes to the IFN-I signature observed in SLE (134, 135). During NETosis triggered by circulating immune complexes in SLE patients there is a production of mitochondrial reactive oxygen species (mito-ROS), dependent on the action of the stress sensor IRE1α (136). Interestingly, inhibition of IRE1 delays the progression of atherosclerosis in the apolipoprotein E-deficient mice and intraperitoneal chronic administration of STF-083010, an inhibitor of IRE1α reduced aorta plaque lesions by 35% (136). NETs are also known to cause vessel occlusion directly, particularly in patients with obesity or cancer (129), a fact that should be kept in mind especially in those SLE patients with a thrombophilic profile. In summary, NETosis appears as an important mechanism linking SLE immunopathogenesis with endothelial damage and CVD.

Monocytes are key players in the early formation of atherosclerotic plaques (137). Activated EC secrete the chemokine CCL2 and attract monocytes to the subendothelial space, where they undergo a differentiation process to foam cells after phagocytizing oxLDL. Through an epigenetic reprogramming, oxLDL-trained monocytes can actively express genes coding for pro-inflammatory proteins, such as TNFα, IL-6, CCL2, and CD36 (138). Total monocyte counts are increased in SLE patients showing clinical and subclinical CVD, but not in patients free of CVD. In 109 patients with disease longer than two years and 31 with earlier disease, total monocyte counts were increased compared to normal controls and patients' monocytes showed a more differentiated pattern, with a higher proportion of intermediate and non-classical monocytes, in direct correlation with higher IL-17 and IFN-I serum levels (139). The inflammatory milieu usually present in SLE patients may induce overactivation of monocytes, and their migration to the intima-media vascular layer, contributing to endothelial dysfunction (139). Monocytes produce intermediate mediators such as ROS and pro-inflammatory cytokines such as TNFα and IL-1β, building up endothelial damage through the vicious cycle of inflammation and oxidative stress. An imbalance within the monocyte subsets defined by their expression of CD14 and CD16, is of relevance in the CV risk of SLE patients. Classical, quiescent, monocytes are strongly positive for CD14, the lipopolysaccharide receptor, but do not express the Fcγ receptor III CD16. In contrast, monocytes co-expressing CD16 and CD14, (intermediate monocytes), and CD14^dim^CD16^+^ (non-classical monocytes) are proinflammatory and pro-atherogenic. They associate with myocardial dysfunction and recovery following MI in the general population, although their relationship to subclinical atherosclerosis is less clearly defined (119). Non-classical CD14^low^CD16^+^ and intermediate CD14^+^CD16^+^ monocytes represent almost 30% of all circulating monocytes and are more differentiated. There are evidence showing a reduction in non-classical monocytes in active SLE patients (140). Mikołajczyk et al, assessed the relationships between the three monocyte subpopulations and IMT in SLE patients. The percentage and absolute numbers of CD14^dim^CD16^+^ or non-classical monocytes positively correlated with IMT in a small cohort of SLE patients (n=42) (119), while the other two subpopulations did not reach a statistically significant difference with healthy controls. It is worth noticing that another study showed augmented amounts of intermediate CD14^+^CD16^+^ monocytes in SLE patients independently of their CV status (139).

Given the pro-inflammatory activity of monocytes and the anti-inflammatory effect of HDL, the monocyte/HDL ratio (MHR) has been proposed as a biomarker of systemic inflammation (141) and has been demonstrated to be a prognostic indicator of CV risk in patients with chronic kidney disease (120). In addition, higher MHR ratios were significantly and independently associated with serum levels of high sensitive C-reactive protein and slow coronary flow (142), and with the severity of CAD in patients with acute coronary syndrome (143). In a cohort of 104 patients with SLE Wang et al. demonstrated higher values of MHR in those patients with carotid atherosclerotic plaques (0.32 ± 0.18 vs 0.26 ± 0.15; p = 0.015), as well as positive correlations of MHR with the carotid IMT (cIMT: r = 0.228; p = 0.001) in patients with SLE (122). Besides MHR, the ratio of CD14^-^CD16^-^ LDG/HDL (nLDR) can also be of value as a biomarker to identify SLE patients with subclinical CVD in the absence of traditional risk factors (139).

The preservation of endothelial integrity depends on the recruitment of sufficient numbers of bone marrow-derived EPC to the site of vascular injury. In addition, functional angiogenic CD3^+^CD31^+^CXCR4^+^CD28^+^ (either CD4^+^ or CD8^+^) T cells (Tang) cooperate with EPC in repairing damaged endothelium (144, 145) through a paracrine effect mediated by the production of multiple proangiogenic cytokines, including VEGF, IL-8, and MMPs. An inverse relationship of Tang cells with age and CVD has been described (144), and they could be useful as new immunological biomarkers for the assessment of CV risk. There is contrasting evidence of the role of Tang cells in patients with SLE, from increased proportions in patients with LN (125), to reduced total numbers but increased percentage of the senescent CD28^-^ subset (126), and no differences with healthy controls (125, 139). The presence of anti-dsDNA autoantibodies may identify a subset of SLE patients associated with increased Tang cells, endothelial damage and higher risk of vasculopathy (125). It is possible that an early phenotypic transition to a CD28^-^ and cytotoxic phenotype may cancel the pro-angiogenic properties of Tang cells and turn them vasculotoxic in SLE patients (126, 139). On the other hand, Th1/Th17 and senescent CD4^+^CD28^-^ T cells cause direct endothelial damage (139). The decreased number of pro-angiogenic EPC and Tang cells found in SLE patients, even in those without CVD, may compromise the repairing of vascular damage caused by the combined effect of LDG, intermediate monocytes, CD4^+^CD28^-^ senescent and Th17 cells. Increased serum VEGF and circulating Tang cells and EPC have been described in patients with LN (124), supporting the hypothesis that Tang cells may play a significant role in the repair of damaged endothelium in SLE patients with renal involvement (124). However, the expansion of an immunosenescent CD28^-^ Tang cell subset with pathogenic potential may also contribute to the enhanced risk of CVD associated to SLE. Further studies are required to clarify the function of CD4^+^, CD8^+^ and CD28^-^ Tang cell subsets, and the net result of their differential expression in SLE patients.

The plasma of SLE patients has atherogenic properties by promoting endothelium damage and accelerating the development of atherosclerosis (146). Normally, the cholesterol-rich VLDL is converted to LDL after undergoing lipolysis in plasma. However, in SLE patients with anti-lipoprotein lipase autoantibodies suppress its lipoprotein lipase activity required to hydrolyze chylomicrons and triglycerides in VLDL, leading to their accumulation in the plasma. In addition, a small dense LDL subtype that undergoes oxidative stress by reactive oxygen species (ROS) in the subendothelial space, is elevated in SLE patients (147, 148), and is able to penetrate easily through the vascular wall and promote atherogenesis (149). Aggregation of immune complexes in blood vessels of SLE patients promotes fixation of the early complement component C1q, followed by upregulation of adhesion molecules and increased monocyte and platelet adherence, leading to endothelial damage (146). Subsequently, monocytes uptake oxLDL and get transformed into foam cells, the building blocks of the fatty streak in the blood vessel intima (146).

EPC generation is the primary endothelial health protection mechanism, by maintaining angiogenesis and preserving the endothelial integrity. IFN-I and other pro-inflammatory factors induce significant impairment in the capacity of EPC to differentiate into mature EC and repair the vasculature. For example, IFN-α down-regulates IL-1β and VEGF, and upregulates IL-18 and its activator caspase-1; IL-1β promotes the differentiation of EPC, whereas IL-18 inhibits the differentiation of EPC. IL-10 inhibits EC differentiation further aggravating the IFNα-mediated EPC dysfunction (150). A reduction of circulating EPC associates with subclinical atherosclerosis in 46 SLE patients. It also correlated with hypertension, tobacco use, insulin resistance, and metabolic syndrome, suggesting that their measurement in peripheral blood could be useful as a biological marker of CVD risk in SLE (118). It should be mentioned that the finding of low proportions of EPC in lupus has not been universally confirmed (149). It is possible that differences in the methods of detection, quantification and identification of EPC and in their correlation with clinical status and treatment protocols of patients might explain these controversial findings.

## Concluding remarks

Higher cardiovascular risk in SLE is a leading cause of death among SLE patients. Classical immune modulatory treatments can regulate atherosclerosis development, supporting a relationship between CVD and chronic inflammation. To date, no drug has proven a preventive activity on atherosclerosis. The recent reports of clinical trials using anti-inflammatory agents suggest that targeting specific inflammatory pathways is a promising opportunity for the prevention and treatment of CVD in SLE (151). Thus, the management of atherosclerosis in SLE patients requires the monitoring of inflammatory activity in addition to classical cardiovascular risk factors. Given the central role of IFN-I in the induction of endothelial damage and plaque formation, the arrival of new drugs addressing the IFN-I pathway to the field of SLE treatment can potentially change drastically the cardiovascular outcome of the patients in a near future. The definition of precise pathogenic immune mediators involved in CVD in SLE will be key in the development of CVD biomarkers in a near future, allowing prevention and early detection of cardiovascular events in SLE patients.

## CYTED RIBLES Network members

Ana María Blasini, Centro Nacional de Enfermedades Reumáticas, Division of Rheumatology, Hospital Universitario de Caracas, and Universidad Central de Venezuela, Caracas, Venezuela. Víctor González-Rumayor, Atrys Health, Barcelona, Spain. Roberto Muñoz-Louis, Hospital Docente Padre Billini, Servicio de Reumatología, Santo Domingo, República Dominicana. Bernardo A. Pons-Estel, Centro Regional de Enfermedades Autoinmunes y Reumáticas (CREAR), Grupo Oroño, Rosario, Argentina. Guillermo Pons-Estel, Centro Regional de Enfermedades Autoinmunes y Reumáticas (CREAR), Grupo Oroño, Rosario, Argentina. Rosana Quintana, Centro Regional de Enfermedades Autoinmunes y Reumáticas (CREAR), Grupo Oroño, Rosario, Argentina. Martín A. Rodríguez, Centro Nacional de Enfermedades Reumáticas, Division of Rheumatology, Hospital Universitario de Caracas, and Universidad Central de Venezuela, Caracas, Venezuela. Manuel F. Ugarte-Gil, Grupo Peruano de Estudio de Enfermedades Autoinmunes Sistémicas. Universidad Científica del Sur, Rheumatology Department, Hospital Nacional. Guillermo Almenara Irigoyen, EsSalud, Rheumatology, Lima, Peru. Gloria Vásquez, Grupo de Reumatología y Grupo de Inmunología Celular e Inmunogenética. Facultad de Medicina, Universidad de Antioquia, Colombia.

## Author contributions

CM coordinated the manuscript. All authors contributed to the article and approved the submitted version.

## Funding

RIBLES Network is funded by Programa Iberoamericano de Ciencia y Tecnología para el Desarrollo (#P220RT0080).

## Acknowledgments

MFU-G (CYTED RIBLES Network member) acknowledges grant research support from Jannsen and Pfizer, unrelated to this manuscript. CM is funded by Nicolás Monardes Programe from Consejería de Salud de la Junta de Andalucía (C2-0002-2019).

## Conflict of interest

GG-M and VG-R (CYTED RIBLES Network member) are employed by the company Atrys Health. CM was affiliated with the GENYO Center, which has a collaboration agreement with Pfizer.

The remaining authors declare that the research was conducted in the absence of any commercial or financial relationships that could be construed as a potential conflict of interest.

## Publisher’s note

All claims expressed in this article are solely those of the authors and do not necessarily represent those of their affiliated organizations, or those of the publisher, the editors and the reviewers. Any product that may be evaluated in this article, or claim that may be made by its manufacturer, is not guaranteed or endorsed by the publisher.
